# Supplementary material for: Measurement accuracy and cutoffs for predicting primary aldosteronism diagnosis using Lumipulse® for renin and aldosterone measurements
Source: PLoS One. 2025 Feb 25;20(2):e0319219. doi: 10.1371/journal.pone.0319219 (PMC11856259; doi:10.1371/journal.pone.0319219)
Supplement: S1 Table — Results are expressed as mean + /- standard deviation; coefficient of variation (CV). (DOCX) [file pone.0319219.s001.docx]

**S1 table. Analytical performances of plasma renin (PRC), plasma aldosterone (PAC) and urinary aldosterone (24UA) with Lumipulse^®^ (Fujirebio^®^) and with reference methods.**

|  | **Intra-assay** | | | | **Inter-assay** | | | |
| --- | --- | --- | --- | --- | --- | --- | --- | --- |
|  | **Lumipulse^®^** | | **Reference methods** | | **Lumipulse^®^** | | **Reference methods** | |
|  | **Mean +/- SD** | **CV (%)** | **Mean +/- SD** | **CV (%)** | **Mean +/- SD** | **CV (%)** | **Mean +/- SD** | **CV (%)** |
| PRC  (mIU/L) | 2.5 +/- 0.2 | 8.6% | 2.5+/-041 | 15.8% | 19.8 +/- 1.3 | 6.7% | 22.2+/-1.2 | 5.5% |
|  | 14.7 +/- 0.3 | 1.8% | 10.0+/-0.5 | 4.9% | 101.6 +/- 4.1 | 4.0% | 100.2+/-3.7 | 3.7% |
|  | 63.3 +/- 3.4 | 5.4% | 87.7+/-1.8 | 2.1% | 1032.1 +/- 35.8 | 3.5% | 169.5+/-8.2 | 4.8% |
| PAC  (pmol/L) | 157 +/- 3.6 | 2.4% | 96.5+/-2.8 | 2.9% | 204 +/- 6.4 | 3.1% | 388 +/- 17.7 | 4.6% |
|  | 351 +/- 4.2 | 1.2% |  |  |  |  |  |  |
|  | 557 +/- 4.2 | 0.7% | 1913+/-19.2 | 1.0% | 1207 +/- 33.6 | 2.8 % | 1871 +/- 113.5 | 6.1% |
| 24UA (nmol/L) | 6.1 +/- 0.11 | 1.7% | 10.8+/-0.27 | 2.5% | 29.7 +/- 1.64 | 5.5% | 24.7+/-19 | 4.8% |
|  | 26.9 +/- 0.17 | 0.6% | 21.4+/-0.50 | 2.32% |  |  |  |  |
|  | 38.6 +/- 0.36 | 0.9% | 39.1+/-.61 | 1.6% | 35.5 +/- 1.44 | 4.1% | 39.8+/-00 | 5.0% |

Results are expressed as mean +/- standard deviation; coefficient of variation (CV).
