# Supplementary figures and images for: Measurement accuracy and cutoffs for predicting primary aldosteronism diagnosis using Lumipulse® for renin and aldosterone measurements
Source: PLoS One. 2025 Feb 25;20(2):e0319219. doi: 10.1371/journal.pone.0319219 (PMC11856259; doi:10.1371/journal.pone.0319219)

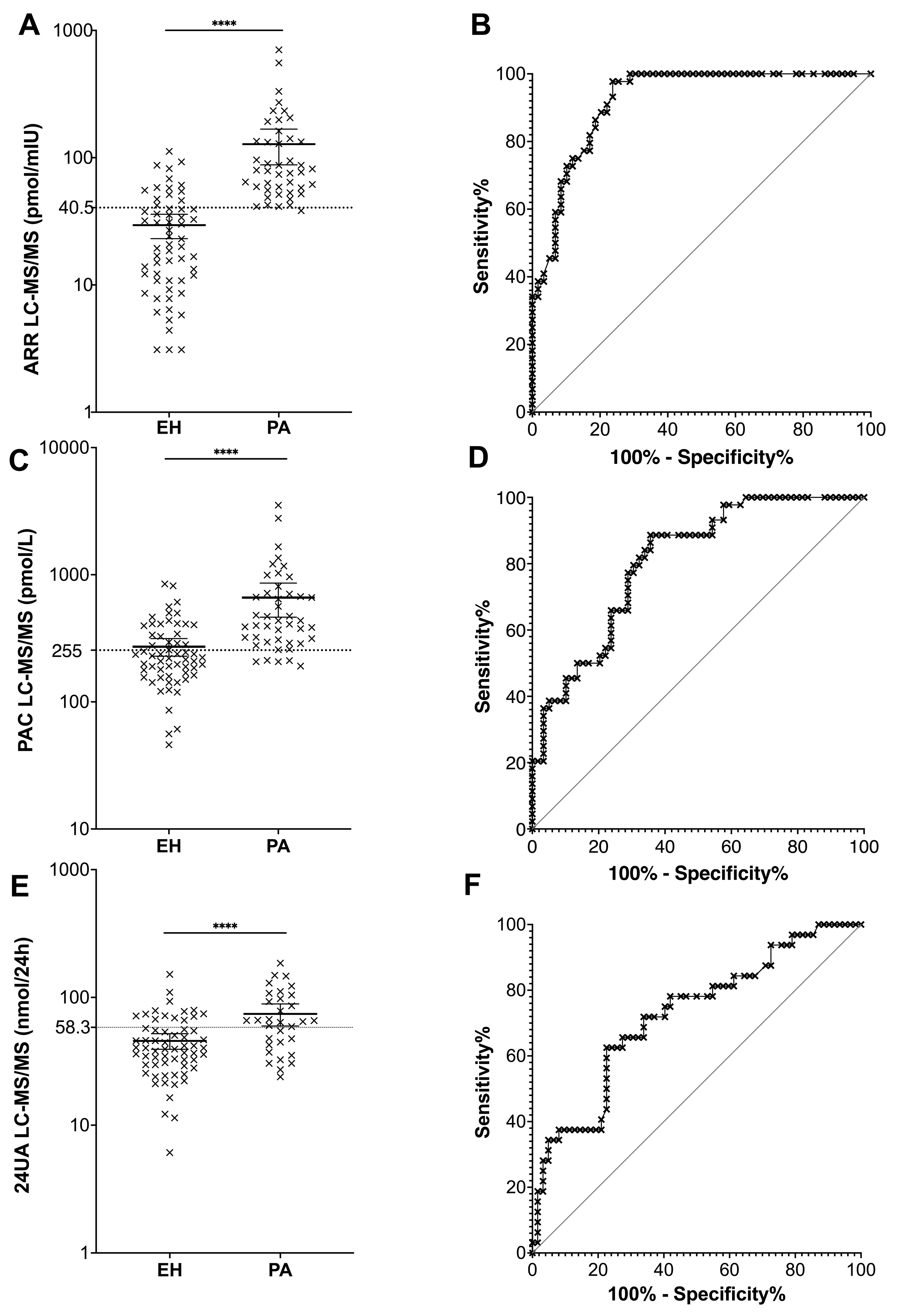

Supplement: S1 Fig — (TIFF) [file pone.0319219.s002.tiff]
