# Supplementary material for: Measurement accuracy and cutoffs for predicting primary aldosteronism diagnosis using Lumipulse® for renin and aldosterone measurements
Source: PLoS One. 2025 Feb 25;20(2):e0319219. doi: 10.1371/journal.pone.0319219 (PMC11856259; doi:10.1371/journal.pone.0319219)
Supplement: Attachment — Submitted filename: renamed_0f66d.docx [file pone.0319219.s003.docx]

We would like to thank the reviewer for the careful review of our manuscript.

As detailed below and in the revised version of the manuscript, we have considered all your suggestions that greatly contributed to improve our manuscript. Below are our responses to your suggestions. We underlined in yellow the part of the manuscript that have been added or rephrased; we barred the part or sentences that have been deleted. All these changes have been highlighted using “Track Changes” option in Microsoft Word.

In addition, supplemental figure 1 has been reuploaded with correction of unit defined in the figure for 24UA expression with LC-MS/MS (nmol/24h instead of µg/d).

Of course, we would be more than glad to answer any additional questions or comments.

Herein, we provide a point-by-point response to the reviewers’ comments.

**Journal Requirements:**

**When submitting your revision, we need you to address these additional requirements.**

**1. Please ensure that your manuscript meets PLOS ONE's style requirements, including those for file naming. The PLOS ONE style templates can be found at**

As requested, we checked that the revised version of our manuscript meets style’s requirement for Plos One manuscript.

**2. Thank you for stating the following financial disclosure: Fujirebio Europe sponsored this study, but did not participate in data analysis nor in writing the manuscript.**

**Please state what role the funders took in the study. If the funders had no role, please state: ""The funders had no role in study design, data collection and analysis, decision to publish, or preparation of the manuscript.""**

**If this statement is not correct you must amend it as needed.**

**Please include this amended Role of Funder statement in your cover letter; we will change the online submission form on your behalf.**

As required, we stated the role of the funder in our study in the conflict-of-interest area of the manuscript. In this way, as suggested, we added lines 389-390 the sentence “This study was sponsored by Fujirebio Europe® but the funders had no role in study design, data collection and analysis, decision to publish, or preparation of the manuscript.”

The cover letter also mentioned the amended role of the Funder as requested with the sentence: “This study was sponsored by Fujirebio Europe, but the funders had no role in study design, data collection and analysis, decision to publish, or preparation of the manuscript”.

**Reviewers' comments:**

**Reviewer's Responses to Questions**

**Comments to the Author**

**1. Is the manuscript technically sound, and do the data support the conclusions?**

**The manuscript must describe a technically sound piece of scientific research with data that supports the conclusions. Experiments must have been conducted rigorously, with appropriate controls, replication, and sample sizes. The conclusions must be drawn appropriately based on the data presented.**

**Reviewer #1: Partly**

**2. Has the statistical analysis been performed appropriately and rigorously?**

**Reviewer #1: I Don't Know**

**3. Have the authors made all data underlying the findings in their manuscript fully available?**

**The PLOS Data policy requires authors to make all data underlying the findings described in their manuscript fully available without restriction, with rare exception (please refer to the Data Availability Statement in the manuscript PDF file). The data should be provided as part of the manuscript or its supporting information or deposited to a public repository. For example, in addition to summary statistics, the data points behind means, medians and variance measures should be available. If there are restrictions on publicly sharing data—e.g. participant privacy or use of data from a third party—those must be specified.**

**Reviewer #1: No**

**4. Is the manuscript presented in an intelligible fashion and written in standard English?**

**PLOS ONE does not copyedit accepted manuscripts, so the language in submitted articles must be clear, correct, and unambiguous. Any typographical or grammatical errors should be corrected at revision, so please note any specific errors here.**

**Reviewer #1: Yes**

**5. Review Comments to the Author**

**Please use the space provided to explain your answers to the questions above. You may also include additional comments for the author, including concerns about dual publication, research ethics, or publication ethics. (Please upload your review as an attachment if it exceeds 20,000 characters)**

**Reviewer #1: This article shows the results of the comparison of measurements of PRC, PAC, and 24-h urine aldosterone excretion (24UA) between Lumipulse^®^ and established reference methods and CVs that indicate measurement accuracy. These results are consistent with those of previous reports. However, a larger sample size may be a strong point. Additionally, the cutoff values for ARR (PAC/PRC), PAC, and 24UA in measurements by Lumipulse^®^, which are optimal for distinguishing between PA and EH diagnosed by confirmatory tests using the gold standard measurement methods, are provided. These data are valuable.**

We thank the reviewer for his carefully reading of our manuscript.

**However, this manuscript has several problems with its main thesis and title that require revisions.**

**Major issues**

**Lines 43–45, 277–278, and 358–359**

**The authors indicate optimal (highest Youden index) cutoffs of ARR, PAC, and 24UA in measurements by Lumipulse® for distinguishing between PA and EH diagnosed by confirmatory tests; however, these are not equivalent to being appropriate cutoffs as screening criteria. Screening aims to identify various suspected patients and is not solely determined by the rate of concordance with the final diagnosis. The cutoff values presented by the authors are just for diagnostic prediction. It would be desirable not to state directly that “these are screening criteria,” but to limit the statement to “the cutoff shows sensitivity of ~~% and specificity of ~~% for PA screening,” as in other reference literatures.**

**The establishment of screening criteria requires consideration of cost-benefit, an acceptable overlook rate, and the boundary between PA and EH that should be set as a borderline between treatment strategies, considering not only confirmatory test values but also complications, disease types, and pathological findings.**

We’d like to thank the reviewer for this suggestion. Indeed, screening criteria must consider both sensitivity of the detection, and cost-benefit consideration. In our study, we considered 3 cut-off values for 3 criteria that must be considered for PA diagnosis, with different sensitivity and specificity performances. Our criteria were defined in accordance with the highest Youden index for each parameter to define an acceptable compromise between sensitivity and specificity.

Indeed, if we focused on sensitivity only, as requested by screening tests, our suggested cutoff values would have been lower. A 100% sensitivity would have led to the use of 19.7 pmol/mIU as cut off value for ARR with 37.3% specificity; 178 pmol/L for 100% sensitivity PAC with 39.0% specificity; and 11.9 nmol/24H for 24UA with 4.8% specificity.

Regarding specifically ARR, our highest Youden index cut off value

Regarding the usual screening criteria ARR. In our study, highest Youden index already reached 95.5% sensitivity, with 67.8% specificity. If we choose to increase sensitivity to 97.7% our cut off value is not significantly different with 31.4 pmol/mIU with 57.6% specificity. If we reached 100% sensitivity, cut off ARR would be 19.7 pmol/mIU with only 37.3% specificity. In this way, regarding ARR our cutoff value with highest Youden index did not differ from cut off value focused on optimal sensitivity. (Table 3)

In this way, we have modified sentence line 43-45 as below to clarify the use of our suggested cutoff values:

“We propose cut-off values of 35 pmol/mIU as the ARR, 260 pmol/L for PAC, and 49 nmol/24h for 24UA for PA diagnosis.

In this way, we also changed the word screening throughout the manuscript to improve clarity of the manuscript.

- Line 74: screening turned into diagnosis
- Line 192: screening turned into diagnosis
- Line 235: screening turned into detection
- Line 243: screening turned into detection
- Line 248: screening turned into detection
- Line 251: screening turned into detection
- Line 257: screening turned into detection
- Line 264: screening turned into detection
- Line 271: screening turned into detection
- Line 275: screening turned into detection
- Line 330: screening turned into detection
- Line 339: screening test turned into PA detection
- Line 341: screening turned into diagnosis
- Line 354: screening PA turned into PA diagnosis
- Line 382: screening PA turned into PA diagnosis

**Moreover, if the initial screening criteria differ among measurement kits, it may cause confusion in screening by general practitioners and field clinics, leading to the avoidance of screening itself. Although the screening criteria for each test kit should be considered, it would be preferable to initially establish uniform screening criteria based on standard measurement methods (LC-MS/MS) and adjust the criteria to match those for each kit.**

As underlined by reviewer, each combination of aldosterone and renin measurements could lead to potent different criteria to diagnose PA. Nonetheless, if LC-MS/MS measurement is considered as the gold standard for aldosterone (plasma and urine) measurement, there is no gold standard for renin measurement even though there is international standardization for aldosterone. Regarding aldosterone measurement, even though LC-MS/MS methods become more and more widespread, plasma aldosterone concentration could differ up to 40% depending on method and calibration (Fanelli et al. 2022). International harmonization of those measurements is yet not achieved. Regarding 24UA, there is currently no consensus regarding cut off value whatever the method in its use in PA diagnosis.

**If the screening criteria are defined based on the sensitivity and specificity of the highest Youden index for each kit, the combination of sensitivity and specificity at the cutoff with the highest Youden index will differ, and the characteristics of each screening criterion will be different. However, some aspects of the present results suggest that lowering the screening criteria would be desirable and such a proposal should be considered.**

We’d like to thank the reviewer for this suggestion. Indeed, we choose to use criteria corresponding to the highest Youden index for each parameter.

Regarding the usual screening criteria ARR, in our study, highest Youden index reached 95.5% sensitivity, with 67.8% specificity. If we choose to increase sensitivity to 97.7% our cut off value is not significantly different with 31.4 pmol/mIU with 57.6% specificity. If we reached 100% sensitivity, cut off ARR would be 19.7 pmol/mIU with only 37.3% specificity. In this context, our cutoff value for ARR with highest Youden index would not differ from cut off value focused on optimal sensitivity (Table 3).

However, regarding PAC, our highest Youden index based cutoff value reach 81.8% sensitivity and 67.8% specificity. If we focused on sensitivity in case of a screening test, to reach 90% sensitivity, PAC cut off value would be 197 pmol/L with only 47.5% specificity. On the other side, if we focused on specificity, 90% specificity would be reached with a cutoff value of 450 pmol/L with only 47.7% sensitivity (Table 4).

Moreover, using the same strategy for 24UA cut off value, our suggested cut off value of 49 nmol/L reached 62.5% sensitivity with 83.9% specificity. If we focused on sensitivity, 90% would be reached with a cutoff value of 22.8 nmol/L24h with only 22.6% specificity; if we focused on specificity, 90% specificity would be reached with cut off value of 57 nmol/24H with only 43.8 sensitivity (Table 5).

As suggested by reviewer, we added this information for each criterion.

- Line 244: A cut off value of 31.4 pmol/mIU would reach 97.7% sensitivity and 57.6% specificity. To reach 100% sensitivity, corresponding cut off value for ARR would be 19.7 pmol/mIU with 37.3% specificity.
- Line 258: To reach 90% sensitivity, PAC cut off value would be 197 pmol/L with 47.5% specificity; while to reach 90% specificity the cut off value would be 450 pmol/L with 47.7% sensitivity.
- Line 272: A 90% sensitivity would be reached with a cutoff value of 22.8 nmol/L24h with 22.6% specificity; a 90% specificity would be reached with cut off value of 57 nmol/24h with 43.8% sensitivity.
- Line 330: A 100% sensitivity would imply a lower ARR cut off value of 19.7 pmol/mIU that would reach only 37% specificity and a very low positive Likelihood ration of 1.6
- Line 353: In our cohort a PAC below 178 pmol/L could exclude PA diagnosis.
- Line 363: In our cohort, a 24UA below 11.9 nmol/24h could exclude PA diagnosis

**Lines 1–2**

**The present study validates the accuracy of Lumipulse® measurement and shows cutoff values with Lumipulse® to predict PA diagnosis by conventional criteria in cases at the author’s institution, but not the diagnostic criteria for PA itself. Therefore, the study’s current title was inappropriate for the content. The title should coincide more with the content of the manuscript, such as “Measurement accuracy and cutoffs for predicting primary aldosteronism diagnosis using Lumipulse® for renin and aldosterone measurements.”**

As suggested by the reviewer, we can improve the readability of our study by changing the title of our manuscript using “Measurement accuracy and cutoffs for predicting primary aldosteronism diagnosis using Lumipulse^®^ for renin and aldosterone measurements”.

**Lines 201–202**

**Of the patients with EH, 22% receive potassium supplementation. What would have caused hypokalemia if not PA?**

We thank the reviewer for this remark. Indeed, 22% of our EH patients received potassium supplementation, while 75% of PA patients did. Nonetheless, EH patients did not exhibit hypokalemia, while PA patients exhibited significantly decreased kalemia despite potassium supplementation. Moreover, as underlined by potassium excretion, EH patients exhibited significantly lower urinary potassium excretion than PA patients.

In our reference center, potassium supplementation was systematically prescribed when patients exhibited a history of acute or chronical, spontaneous or diuretic-induced hypokalemia prior to primary aldosteronism screening (in accordance with International Guidelines (Funder, 2016).

In this way, we added line 208:

“Potassium supplementation was systematically prescribed when patients exhibited any history of hypokalemia in accordance with guidelines [7].”

**Lines 130–132 and 136–137**

**It is stated that only samples from patients without drugs interfering with the RAAS were used. What are the drugs that you defined as precisely interfering with the RAAS?**

As noticed by reviewer, RAAS interfering treatment were discontinued before blood sampling if used. To clarify this point, sentence beginning line 132 has been added for plasma criteria:

“Beta-blockers, angiotensin II type 1 receptor blockers, angiotensin-converting enzyme inhibitors and diuretics were discontinued 2 weeks before blood collection; mineralocorticoid receptor antagonists and renin inhibitors 6 weeks before. Calcium antagonists, α-blockers, and centrally acting antihypertensive drugs were prescribed to patients with severe hypertension if necessary.”

In the same way, sentence beginning line 141 has been changed in urinary criteria section:

“For PA diagnosis, only samples from patients without drugs interfering with RAAS (as described previously) were included (i.e., n=32 PA and n=62 EH).”

**Lines 184–185**

**While Lumipulse® and LC-MS/MS provided similar values for PAC, why does Lumipulse® show smaller values for urinary aldosterone than LC-MS/MS?**

We thank the reviewer for this interesting question. Indeed, we do observe lower urinary aldosterone concentrations with Lumipulse^®^ than with our LC-MS/MS. Fujirebio method is standardized against CRM 6402-a while our LC-MS/MS is standardized using Cerilliant^®^ certified reference material.

However, there are very few studies focusing on urinary aldosterone measurement, even less focusing on method comparison. Nonetheless, it appears to be a general trend that immunoassays urinary aldosterone measurement are lower than those obtained with LC-MS/MS (Ozeki et al. 2022, Bekkach et al. 2016).

All immuno-assay based urinary aldosterone measurement used similar sample preparation for acid hydrolysis of aldosterone glucuronide metabolites. Briefly, all manufacturers’ guidelines recommends urine sample acid hydrolysis using HCl 0,2 N (2:1 HCl:urine) ratio for 20 hours at 30°C Ozeki et al. 2022, Bekkach et al. 2016, Lin et al. 2017). LC-MS/MS method used slightly different acid hydrolysis with increased chlorohydric acid concentration and room temperature hydrolysis (0,6 N 1:1). The major role of hydrolysis condition has been underlined in Yazdanpanah et al. study (Yazdanpanah et al 2023). Using the same LC-MS/MS assay, measurement of urinary aldosterone concentration varies up to 30% depending only on hydrolysis conditions. Yin et al. also showed the importance of acid hydrolysis conditions in pH1 urinary aldosterone measurement (Yin et al. 2019).

This sample preparation could be responsible for the difference observed between Fujirebio and in house LC-MS/MS urinary aldosterone measurement.

In this way, paragraph beginning line 365 has been changed in the discussion section:

“As expected, Lumipulse^®^ 24UA had lower values than LC-MS/MS methods as already showed in previous studies [40, 41]. These lower values are observed with most imuno-assays than with LC-MS/MS. This discrepancy, up to 30%, could be related to the diversity of acid hydrolysis conditions rather than discrepancies in calibrators choice [37, 42]. A few studies have reported a reference interval for 24UA using immune assay methods”.

**Minor issues**

**Line 104**

**Is the unit of ARR not pmol/mIU?**

Thanks for this correction. Indeed, unit must be pmol/mIU instead of pmol/L. It has been changed accordingly line 104.

“An ARR higher than 46 pmol/mIU on at least two different occasions and a baseline PAC in the seated position higher than 360 pmol/L on at least two different occasions confirmed the diagnosis of PA [9]”

**Lines 192–193**

**Why did only 11 of the 18 unilateral PA patients undergo surgery?**

Indeed, among the 18 patients with unilateral PA, 5 of them did not have follow-up in our reference center, one patient finally refuses surgery, and one patient passed away before adrenalectomy. This information was briefly added line 200.

“Among the 7 other patients, 5 of them did not have follow up in our center, one passed away prior adrenalectomy and the last one finally refused surgery.”

**Lines 203–204, Tables**

**The letter “p” is inconsistently expressed in lower and upper case. The significant digits of the p-values are not consistent throughout the text and tables and vary from <0.05–<0.0001.**

We’d like to thank the reviewer for p case. Indeed, p lines 160 and 210 has changed into lower case.

Regarding, significant digits in p values, it has been changed accordingly to reviewer comment throughout the manuscript: in tables 1 and 2, and in the text:

Line 209: “Compared with EH patients, PA patients had a lower plasma potassium concentration (p=0.0018) and PRC (p<0.0001) and a higher PAC, ARR, 24UA, and 24-hour urine potassium excretion (p<0.0001).”

Line 226: “PA patients had a lower PRC (p=0.0005), plasma potassium concentration (p=0.0151), and CKD-EPI eGFR (p=0.0277) and a higher DBP (p=0.0039), PAC, ARR, 24UA, and 24-hour urine potassium excretion (p<0.0001).”

**Lines 329–333**

**The authors presented a cutoff of 260 pmol/L as the boundary between EH and PA, whereas the value of 277 pmol/L presented by Ono et al. was the cutoff used to distinguish APA from IHA and EH. Were these results consistent?**

As underlined by reviewer, Ono et al. focused on the distinction between APA and non-APA patients (both BHA and EH patients), while our study aimed at distinguishing PA patients (both APA and BHA) from EH patients. Moreover, Ono et al. cohort exhibited a higher size and was exclusively Japanese while our cohort was predominantly Caucasian. The Asian population has been shown to have a higher incidence of APA with KCNJ5 mutations (Shimamoto et al. 2014; Nishikawa et al., 2011), and higher PAC values than other PA patients.

In Ono et al., PAC mean was significantly higher in APA patients (n = 133) with mean PAC 888 pmol/L, while PAC were not different between EH (n = 111) and BHA (n = 100) patients with both mean PAC 230 pmol/L.

In our study, 18 patients were APA (mean PAC 968 pmol/L) and 20 BPA (mean PAC 397 pmol/L); in EH patients (n=59) PAC mean was 272 pmol/L. Thus, in our cohort, BPA patients exhibited significantly higher PAC than EH patients, contrary to Ono et al. study. In our cohort,

In this way, both because of the methodological choice to diagnose APA only in Ono et al. and because of the two different population in the 2 cohorts, cut off value for PAC are slightly different.

Paragraph beginning line 347 has been changed accordingly:

“In a Japanese cohort of hypertensive patients, Ono et al. reached 85% sensitivity and 75.8% specificity with a cut off value of 277 pmol/L for PAC to distinguish APA from non-APA (both BAH and EH patients) [16]. In our study, such cut off would slightly decrease sensitivity of PA detection (72.7%) without improvement of specificity, because of the choice to diagnose only APA, with a potent highest proportion of KCNJ5 mutated APA as commonly observed in Asian cohorts.”
